# Thrombosis in vasculitis: from pathogenesis to treatment

**DOI:** 10.1186/s12959-015-0047-z

**Published:** 2015-04-16

**Authors:** Giacomo Emmi, Elena Silvestri, Danilo Squatrito, Amedeo Amedei, Elena Niccolai, Mario Milco D’Elios, Chiara Della Bella, Alessia Grassi, Matteo Becatti, Claudia Fiorillo, Lorenzo Emmi, Augusto Vaglio, Domenico Prisco

**Affiliations:** Department of Experimental and Clinical Medicine, University of Florence, L.go G. Brambilla, 3, 50134 Florence, Italy; SOD Interdisciplinary Internal Medicine, Center for Autoimmune Systemic Diseases–Behçet Center and Lupus Clinic–AOU Careggi Hospital of Florence, Florence, Italy; Department of Experimental and Clinical Biomedical Sciences, University of Florence, Florence, Italy; Nephrology Unit, University Hospital of Parma, Parma, Italy

**Keywords:** Inflammation-induced thrombosis, Thrombo-embolic disease, Deep vein thrombosis, ANCA associated vasculitis, Large vessel vasculitis, Behçet syndrome

## Abstract

In recent years, the relationship between inflammation and thrombosis has been deeply investigated and it is now clear that immune and coagulation systems are functionally interconnected.

Inflammation-induced thrombosis is by now considered a feature not only of autoimmune rheumatic diseases, but also of systemic vasculitides such as Behçet’s syndrome, ANCA-associated vasculitis or giant cells arteritis, especially during active disease.

These findings have important consequences in terms of management and treatment. Indeed, Behçet’syndrome requires immunosuppressive agents for vascular involvement rather than anticoagulation or antiplatelet therapy, and it is conceivable that also in ANCA-associated vasculitis or large vessel-vasculitis an aggressive anti-inflammatory treatment during active disease could reduce the risk of thrombotic events in early stages.

In this review we discuss thrombosis in vasculitides, especially in Behçet’s syndrome, ANCA-associated vasculitis and large-vessel vasculitis, and provide pathogenetic and clinical clues for the different specialists involved in the care of these patients.

## Introduction

The relationship between inflammation and thrombosis is not a recent concept [1], but it has been largely investigated only in recent years [2]. Nowadays inflammation-induced thrombosis is considered to be a feature of systemic autoimmune diseases such as Systemic Lupus Erythematosus (SLE) [3], Rheumatoid Arthritis (RA) [4], or Sjögren Syndrome (SS) [5]. Moreover, both venous and arterial thrombosis represents a well known manifestation of Behçet syndrome (BS) [6]; more recently accumulating data have demonstrated a significant increase in thrombo-embolic events both in ANCA-associated vasculitis (AAV) and large-vessel vasculitis (LVV) [7], especially during active disease. These findings have important consequences in terms of management and treatment; for example, BS requires immunosuppressive treatment rather than anticoagulation for venous or arterial involvement [8], and perhaps one might speculate that also in AAV or LVV an aggressive anti-inflammatory treatment during active phases could ameliorate vascular involvement especially in early stages.

Here we will highlight some of the main pathogenetic and clinical aspects of thrombosis in systemic vasculitis, and in particular in BS, AAV and LVV [Table 1].

**Table 1:** **Summary of clinical and therapeutic features of thrombotic events in systemic vasculitis**

***Disorders***	***District involvement and treatment***	***Data based on***	***References***
**Behçet’s syndrome**	***Venous involvement -*** venous involvement is common and makes up 75% of all vascular complications. Venous thrombosis occurs more frequently in males with active disease during the early years, sometimes at the onset of disease, and tends to recur.	Large survey (387 pts) and retrospective evaluation (2319 pts)	[6,38-41]
	***Arterial involvement -*** the most characteristic arterial manifestations are aneurysms whereas arterial thrombosis is less common. The coexistence of thrombosis and aneurysms is a peculiar feature of Behçet.	Retrospective evaluations and expert experience	[6,11,13,42-44]
	***Treatment -*** the management of vascular thrombosis is based on immunosuppressants rather than anticoagulants. Azathioprine and cyclosporine in association with low dose corticosteroids are usually the first choice in the treatment of deep vein thrombosis and superficial vein thrombosis, while cyclophosphamide is the suggested treatment for arterial involvement. In resistant cases anti-TNFα agents could be considered.	European League Against Rheumatism recommendations, large monocentric experience (64 pts)	[8,13,45-51]
**ANCA-associated vasculitis**	***Venous involvement -*** increased incidence of venous thromboembolism, especially during active disease.	Multicentric randomized placebo-controlled trial (180 pts), retroprospective analysis (up to 1130 pts) and monocentric experience (19 pts)	[61,69-74]
	***Arterial involvement -*** increased incidence of arterial involvement and particularly of cardiovascular events.	Two large retrospective studies (113 and 239 pts respectively)	[75-77]
	***Treatment -*** there are controversial data on the use of statins, while there are no significant data on the use of antiplatelet and/or anticoagulant therapy.	In *vitro* study, case reports	[78-80]
**Large-vessel vasculitis**	***Venous involvement -*** increased risk of venous thromboembolism, both deep vein thrombosis and pulmonary embolism, in particular during the first year after diagnosis. Similar data are reported in polymyalgia rheumatica.	Large population-based study (909 pts) and nationwide population study (535.538 individuals)	[87-90]
	***Arterial involvement -*** increased risk of cardiovascular events, especially in giant cell arteritis.	Large cohort study (3500 pts) and retrospective analysis (210 pts)	[91-100]
	***Treatment -*** the use of antiplatelet/anticoagulant therapy is not effective for primary prophylaxis, whilst it could be beneficial as combination therapy with corticosteroids in established giant cell arteritis. In Takayasu disease the use of antiplatelet treatment could be protective for ischemic events.	Cumulative meta-analysis (6 retrospective studies, 914 pts), monocentric retrospective evaluation (48 pts), retrospective analysis (297 pts)	[101-104]

### Search strategy and selection criteria for review

We searched Pubmed matching the key search terms “thrombosis in vasculitis”, “Behçet and thrombosis”, “ANCA-associated vasculitis and thrombosis”, “Large vessel vasculitis and thrombosis”. Full texts, as well as abstracts of published articles were reviewed. The search was limited to papers published in English language, and was conducted through December 2014.

### Behçet’s syndrome

#### Introduction

Behçet’s syndrome is a systemic vasculitis with a heterogeneous clinical phenotype [9], characterized by oral and genital ulcerations, uveitis, skin lesions and vascular, neurological and gastrointestinal involvement. International diagnostic criteria for BS, first published in 2006 and recently revised [10], have included vascular involvement as a diagnostic criterion. The term angio-Behçet is used to define patients in whom large vessel lesions are the main feature. Both arterial (e.g. aneurysms) and venous involvement (e.g. deep venous thrombosis) can occur [11]. A peculiar feature of BS is the association between venous and arterial damage; some authors have reported that pulmonary artery aneurysms and peripheral venous involvement coexist in up to 90% of the patients [12].

#### Pathogenesis of (athero)thrombosis in Behçet’s syndrome

The pathophysiology of thrombosis in Behçet’s syndrome (BS) is not well known, but systemic inflammation seems to play a major role whereas other thrombophilic factors are less relevant [13]. However, it should be underlined that inflammation and haemostasis are closely linked and that the immune system plays a role in the thrombotic process [14]; BS may thus be considered a model of inflammation-related thrombosis [15].

- Immune system

A generalized derangement of CD4+ lymphocytes, monocytes and neutrophils and an overproduction of proinflammatory cytokines related to Th1 cells, such as interferon-gamma (IFNγ), tumor necrosis factor (TNF)α, interleukin (IL)1, IL6, IL8 and IL12, have been observed in BS [16]. Th17 cells along with their cytokines, IL17A, IL22, TNFα, also seem to be involved in the inflammatory process, and so is IL21 which may promote Th1 and Th17 differentiation and Treg cells suppression [17]. This condition is able to self-renew, so amplifying the proinflammatory environment and promoting a prothrombotic state. Different mechanisms of inflammation may affect endothelial cells; in particular, in BS anti-endothelial cell antibodies (AECA) have been described as a possible link between immune response and endothelial dysfunction [18,19].

b) Coagulation system

In BS, the coagulation system may promote inflammation and thrombosis through multiple factors such as the tissue factor (TF) pathway, thrombin and the protein C system along with an impaired fibrinolysis [13]. Endothelial cell dysfunction, resulting from immunological and inflammatory factors, seems to be a characteristic feature of BS and plays a key role in the pathogenesis of thrombotic manifestations. A decreased production of nitric oxide (NO), a prominent marker of endothelial dysfunction, was reported in some patients with active BS [20], and interestingly a reduction in asymmetric dimethylarginine, the endogenous inhibitor of NO synthase, has also been observed [21]. Moreover, high levels of other endothelial injury markers, such as circulating von Willebrand factor [22] and thrombomodulin [23] were found in patients with active BS. Increased serum levels of vascular endothelial growth factor (VEGF), a marker of angiogenesis, and of some adhesion molecules such as intercellular adhesion molecule 1 and E-selectin, produced by activated endothelial cells, were reported in BS patients. These markers were increased particularly during the active stage of the disease, thus underlying the close relationship between endothelial cells, leukocytes and autoimmune mechanisms [24]. Another adhesion molecule, P selectin, was found to be elevated in the plasma of BS patients. This molecule, located in the Weibel-Palade bodies of endothelial cells and in the granules of platelets and released into the plasma during platelet activation, promotes inflammatory reactions by facilitating leukocyte recruitment at the site of injury [25]. Some studies have also reported signs of enhanced platelet activation including higher concentrations of platelet microparticles (MPs) in BS patients compared with healthy controls [26]. Moreover, several studies have reported high plasma levels of homocysteine in BS patients with a history of thrombosis, especially in the active phase of disease [27], while data are conflicting on the possible correlation between homocysteine and HLA-B51 [28]; endothelial function, tested by flow-mediated dilatation of the brachial artery was found to be significantly impaired in BS patients [29]. Different haemostatic factors have been investigated in BS with discordant results. Controversial data were reported about the role of some procoagulant factors, such as coagulation factor V G1691A (factor V Leiden mutation) and prothrombin G20210A polymorphisms in BS, suggesting that they might be an additional risk factor for thrombosis in certain populations. Factor V Leiden mutation is reported to be more prevalent in Turkish [30,31], but not in Italian, Spanish and Israelian patients [32-34]. Prothrombin gene mutation was not reported to be relevant in several studies [35], but a meta-analysis showed a significant association between the presence of prothrombin G20210A mutation and thrombosis in BS, when Turkish patients were excluded [33]. Instead, deficiencies of natural anticoagulant proteins including protein C, protein S and antithrombin have not been associated with thrombosis in BS patients [36]. High levels of Lipoprotein(a) were found in BS patients and might be involved in the pathogenesis of thrombosis by impairing fibrinolysis [15]. Furthermore, high plasma levels of thrombin-activable fibrinolysis inhibitor were reported in BS patients which could result in a significant reduction of clot lysis process [37].

#### Venous thrombosis in Behçet’s syndrome

Thrombosis is the most frequent vascular manifestation in BS patients, its prevalence ranges from 14% to 39% and venous involvement is characteristically more common and makes up 75% of all vascular complications [38]. Venous thrombosis occurs more frequently in males with active disease during the early years, sometimes at the onset of disease, and tends to recur [39,40]. Deep vein thrombosis (DVT) and superficial vein thrombophlebitis (SVT) of lower extremities are the typical manifestations, but thrombosis may occur anywhere in the venous system and the involvement of atypical sites such as hepatic veins, superior and inferior vena cava and cerebral sinus veins are also observed [6]. BS should be always considered in the differential diagnosis of venous thrombosis in unusual sites in young individuals.

In some studies SVT have been reported as the most frequent lesions. It usually occurs in the lower limbs as painful nodules similar to erythema nodosum, but in some cases it may be a complication of venipuncture reflecting a pathergy-like effect in the venous wall [41].

#### Arterial involvement in Behçet’s syndrome

Arterial involvement is present between in 1 to 7% of the patients [6]. The most characteristic arterial manifestations in BS patients are aneurysms whereas arterial thrombosis is less common [11]. These complications may remain asymptomatic or result in life- or organ-threatening infarctions such as acute myocardial infarction, stroke, intestinal infarction, intermittent claudication or gangrene of the lower extremities [13]. Arterial occlusions and venous thromboses sometimes coexist in the same patient and may be associated with aneurysms [42,43]. Thus, the coexistence of thrombosis and aneurysms is a peculiar feature of BS. Overall, cardiac manifestations are rare in BS patients, with a reported incidence between 1% and 6%, and are mainly represented by intracardiac thrombosis and coronary artery disease [44].

#### Treatment

Currently, the management of vascular thrombosis in BS patients is based on immunosuppressive therapy to reduce the inflammation of the vessel wall. Anti-inflammatory treatments are able to promote a rapid and effective regression of the vascular lesions, to prevent the extension of thrombosis and its recurrence. The European League Against Rheumatism (EULAR) recommendations suggest immunosuppressive treatment with agents such as corticosteroids (CS), azathioprine (AZA), cyclophosphamide (CYC) or cyclosporine A (CsA) [8].

AZA and CsA in association with low dose CS are usually the first choice in the treatment of DVT and SVT. In some serious cases such as Budd-Chiari Syndrome or superior and inferior vena cava thrombosis, treatment with pulse CYC is suggested [45]. CYC is also the recommended treatment in BS patients with arterial involvement.

Usually, anticoagulants alone are not recommended in BS patients [8]. Actually, only for CNS venous thrombosis some authors suggest anticoagulation, with or without corticosteroids [46,47]. The pathophysiology of thrombosis in BS, where systemic inflammation promotes the prothrombotic state leading to the formation of a thrombus tightly adherent to the vessel wall with a low rate of embolism [13], the discordant data on coagulation abnormalities, the possibility of the coexistence of PAA and thrombosis and the low efficacy of the anticoagulants reported in several studies are the main reasons that support the treatment with immunosuppressive agents and not with anticoagulants in BS patients. However, the role of anticoagulants continues to be an open question.

Sometimes thrombosis in BS as well as other manifestations are refractory to traditional immunosuppressive therapy and tend to recur, so more effective therapeutic options are required. According to the hypothesis that inflammatory cells and proinflammatory cytokines, including gamma-delta T cells (γδ T cells) and TNFα, play a major role in the development of thrombosis [48], a successful use of anti-TNFα agents, especially for uveitis, neurological and gastrointestinal manifestations, has been increasingly reported in BS patients [48].

Cases of angio-Behçet patients resistant or intolerant to conventional immunosuppressive therapy successfully treated with anti-TNFα agents have been increasingly reported over the last years. However, the experience with anti-TNFα agents for major vessel involvement is limited to case reports. An analysis of 369 BS patients treated with anti-TNFα agents in 20 different countries has recently been reported [49], but only few cases of treatment with anti-TNFα agents in BS patients with vascular complications have been described [50,13].

Interestingly, among conventional agents used in cardiovascular diseases, only atorvastatin and lisinopril have been investigated, with results showing a possible improvement in endothelial function in BS patients [51].

### ANCA-associated vasculitis

#### Introduction

Anti-neutrophil cytoplasmic antibody (ANCA)-associated vasculitis (AAV) comprises a group of disorders characterized by necrotizing inflammation of small vessels and the presence of ANCA directed to specific antigens, particularly proteinase 3 (PR3-ANCA) and myeloperoxidase (MPO-ANCA) [52]. The main clinical entities within the AAV spectrum are Granulomatosis with Polyangitiis (GPA, formerly Wegener’s Granulomatosis), Microscopic Polyangitiis (MPA) and Eosinophilic Granulomatosis with Polyangitiis (EGPA, formerly Churg-Strauss syndrome) [53].

#### Pathogenesis of (athero)thrombosis in ANCA-associated vasculitis

- Immune system

Endothelial cell dysfunction is a feature of AAV and is probably caused by the interaction between neutrophils (activated by TNFα and ANCA) and endothelial cells, with consequent massive oxidative stress finally leading to atherothrombotic complications [54].

Recently, an additional mechanism of neutrophil activation has been described, termed NETosis; neutrophils are able to release extracellular nucleic acids associated with histones and granular proteins capable of entrapping bacterial agents. These neutrophil extracellular traps (NETs) have been also implicated in thrombotic events and seem to be a potential bridge between autoimmunity and coagulation. In particular, neutrophils primed by ANCA degranulate and release NETs, which in turn contain MPO and PR3, that act as autoantigens, thus creating a self-amplyfing process [55]. In active AAV, neutrophils release high levels of TF-expressing NETs [56]; moreover NETs are able to promote thrombosis by inhibiting the TF pathway inhibitor and by recruiting platelets [14]. Finally, an intriguing *in vivo* model in which dendritic cells primed by NETs are able to induce the production of ANCA in mice has been recently proposed, thereby strengthening the role of NETs in AAV [57].

MPs are involved in many biological mechanisms, including thrombosis [58]. Neutrophil-derived MPs have been recently demonstrated in active AAV [56]; they contain inflammatory mediators such as platelet activating factor, adhesion molecules and MPO, suggesting that they may activate and damage endothelial cells [59,60].

In *vitro* and in *vivo* models have shown also in AAV a possible role of AECA in inducing endothelial cell dysfunction via an antibody-dependent cytotoxicity mechanism [18,19].

In EGPA, in addition to neutrophils, also eosinophils seem to promote vascular injury via the release of preformed granules during active disease. In particular eosinophil cationic protein and membrane basic protein can inhibit the activation of the protein C system and, at the same time, induce platelets to produce platelet factor 4, able to impair heparin function. TF and eosinophil peroxidase are also released by activated eosinophils; the former activates factor X, while the latter induces endothelial cells to express TF [61-63].

b) Coagulation system

Plasminogen has been described as an autoantigen in PR3-ANCA patients, and its interaction with autoantibodies directed towards complementary PR3, a recombinant protein translated from the antisense strand of PR3 cDNA, is able to block its conversion to plasmin, ultimately impairing fibrinolysis [64,65].

Boomsma et al. have shown that elevated levels of soluble thrombomodulin and plasma endothelial protein C receptor, which are markers of endothelial cell damage were increased in GPA patients and partly correlated with disease activity [66].

Patients with GPA have not been reported to have an increased prevalence of thrombophilic factors such as Factor V Leiden and prothrombin gene mutations, while they were found to have an increased prevalence of anticardiolipin antibodies (aCL), although no correlation with thrombotic events was reported [67].

More recently a procoagulant state was reported in non active AAV as well: endogenous thrombin potential and Factor VIII were found to be increased in patients in stable remission compared to healthy controls [68].

#### Venous thrombosis in ANCA-associated vasculitis

In recent years, evidence supporting an increased frequency of venous thrombotic events in AAV has arisen. The prevalence of venous thrombosis in AAV ranges between 5.8% and 30% [61]. Relevant data came from the WGET trial (Wegener’s granulomatosis Etanercept Trial) published in 2005 [69]; in this study 180 patients with GPA followed for more than 2 years had an increase incidence of venous thromboembolism (VTE), especially during active disease. Subsequent studies [70-72] confirmed a high frequency of thrombotic events among patients with AAV, especially during early and active disease stages. In a recent Australian case series of EGPA patients [73] an increased incidence of VTE (both in typical and atypical sites) and pulmonary embolism (PE) was reported. Very recently a retrospective study conducted in a Tertiary Reference Center in Denmark has confirmed that patients diagnosed as having GPA have a significant risk of VTE both early and late during the course of their follow-up (median 7.2 yrs) and are hospitalized several times for PE and DVT [74].

#### Arterial involvement in ANCA-associated vasculitis

An increased frequency of arterial events in AAV has been reported in the literature. The estimated prevalence of arterial involvement in AAV is between 3.1% and 18.7% [75,76]. In a retrospective study, 113 patients with AAV were compared with a matched population with non-inflammatory chronic kidney disease, showing a significant increase in cardiovascular events (CVE) in the AAV group. Previous cardiovascular disease, dialysis dependence, and smoking were the strongest predictors of CVE. Of note, only 2 patients with EGPA were included, while the majority were patients diagnosed as GPA (n = 65) and Microscopic Polyangiitis (MPA, n = 46) [75]. Another retrospective study was conducted using the Danish National Hospital Register, on 293 patients with GPA; an increased risk of acute myocardial infarction was observed, in particular in men aged >50 yrs at the time of diagnosis and with a cumulative dose of cyclophosphamide >36 grams. Interestingly, this GPA population had an increased risk of CVE both in the early (within 5 years of diagnosis) and in the late (after 10 years of diagnosis) phase of the disease, suggesting that not only acute, but also chronic inflammation may be implicated in this process [76].

In 2011 a prognostic tool to define the 5-year cardiovascular risk was created for AAV patients based on data from four European Vasculitis Study Group (EUVAS) trials of WG and MPA considering a total population of 535 patients. The results indicated that almost 12% of newly diagnosed GPA and 16% of MPA patients had presented at least one CVE, defined as cardiovascular death, myocardial infarction, coronary artery bypass graft/percutaneous coronary intervention or stroke. Interestingly in this risk algorithm, while an older age and the presence of diastolic hypertension were associated with an increased incidence of CVE, the positivity of PR3-ANCA was associated with a lower cardiovascular risk [77].

#### Treatment

An *in vitro* model has demonstrated that simvastatin is able to significantly inhibit neutrophil degranulation induced by ANCA [78], so suggesting its potential role in clinical practice beyond cardiovascular protection, although some cases of AAV induced by statins have been reported [79,80]. Currently there are no significant data on the use of antiplatelet and/or anticoagulant therapy in AAV.

### Large vessel vasculitis

#### Introduction

Large vessel vasculitis (LVV) usually comprises Giant Cell Arteritis (GCA) and Takayasu arteritis (TA) [52]. The histopathologic features of these two entities are similar, whilst they substantially differ in the age range of the affected patients, since TA usually affects young women and GCA predominates in the elderly [52].

#### Pathogenesis of (athero)thrombosis in large vessel vasculitis

The pathogenesis of atherothrombotic events in these inflammatory conditions is overlooked.

- Immune system

As in BS and in AAV, even in LVV, and especially in TA, a possible role of AECA in inducing endothelial damage has been suggested [18,19].

More interestingly, one of the possible vascular complications is the development of aneurysms as a consequence of inflammatory damage. Vessel wall remodeling in LVV starts from the adventitial layer, with an infiltrate mainly consisting of Th1/Th17 lymphocytes activated by resident dendritic cells [81], and macrophages producing pro-inflammatory cytokines such as IL1β and IL6; macrophages of adventitial and medial layers are responsible for the production of growth factors, such as platelet-derived growth factor and VEGF, which induce intimal hyperplasia [81,82]. Interestingly the innate immunity also contributes to vascular remodeling, via pentraxin 3 (an innate pattern recognition receptor), which accumulates at the site of active remodeling both in GCA and TA vessels [83,84].

b) Coagulation system

Inheritable thrombophilia does not seem to have any role in GCA patients; a high prevalence of antiphospholipid antibodies was observed, without any correlation with vascular events [85]. High levels of homocysteine were reported in a single study in patients with polymyalgia rheumatica (PMR) and GCA, probably related to corticosteroid treatment [86].

#### Venous thrombosis in large vessel vasculitis

Venous thrombosis in LVV has been poorly investigated. In GCA the incidence rate of venous involvement is estimated to be 13.3/1000/year for VTE and 8.5/1000/year for DVT [87]. A recent population-based study has tried to fill this gap; in a large cohort comprising 909 patients with GCA, an increased risk of VTE (both DVT and PE) in particular during the first year after diagnosis has been observed [87]. Interestingly, similar findings were reported in a recent Swedish nationwide hospital-based study in patients affected by PMR, a condition strictly related to GCA, who showed an increased incidence of PE. Also in this population the risk was higher during the first year after diagnosis, suggesting a possible role of inflammation in the pathogenesis of the vascular events [88]. None of the traditional risk factors has been definitely linked so far to an increased occurrence of venous events in GCA; in two previous reports of small populations, a role for aCL antibodies was hypothesized [89,90].

#### Arterial involvement in large vessel vasculitis

Interesting data about arterial involvement in LVV are available. A recent cohort study evaluating nearly 3500 patients with GCA has reported an increased risk of CVE, especially in the first month after the diagnosis; the study was mainly limited by the source of data (primary care database) and the lack of data about temporal artery biopsies [91].

The influence of the traditional cardiovascular risk factors in GCA is far from being established, however a retrospective Spanish study has reported several risk factors for atherosclerosis at the time of diagnosis, and among them, especially hypertension significantly enhances the risk of developing severe ischemic events [92].

Another Spanish study on 287 GCA patients reported that stroke occurred in almost 3% of them, mostly within 1 month of diagnosis; most of the patients were male, smokers and with arterial hypertension, and permanent visual loss was the best predictor of stroke occurrence [93]. A recent population-based study has confirmed these data, except for a higher incidence of stroke (7%) [94].

An Italian study conducted by Salvarani et al. demonstrated that the PlA2/A2 homozygosity of the GPIIIa gene is associated with anterior ischemic optic neuritis in GCA and could partly explain the reduced capacity of aspirin to prevent cerebral events in this population [95]. Another study by the same group [96] interestingly showed that, together with a history of arterial hypertension and previous CVE, also low levels of acute phase reactants were associated with occurrence of cerebral accidents; this evidence is not surprising, since the data published by Hernández-Rodríguez and colleagues demonstrated that the angiogenic properties of IL6 could compensate ischemic injury in GCA patients [97]. Curiously, a recent retrospective study on 245 patients with GCA has shown that the risk of acute coronary syndromes in patients with GCA was comparable with that of non-GCA patients and that some cardiovascular risk factors, such as diabetes, were found to be protective in the GCA population [98]. As previously reported, GCA patients with coronary artery disease however demonstrated an increased risk of aortic aneurysm and arterial thrombosis [99].

In a Korean retrospective study on Takayasu arteritis, 21 of 190 (11%) patients, almost all young (mean age <40 years) female had presented a stroke [100]. In this population, risk factors were comparable between Takayasu-stroke and Takayasu non-stroke patients, while transient monocular blindness (considered a warning bell of cerebral events) occurred at low frequency.

#### Treatment

A recently published comprehensive meta-analysis has clearly shown that in GCA the use of antiplatelet/anticoagulation therapy is not effective for primary prophylaxis, whilst it could be beneficial as combination therapy with corticosteroids in established GCA, without an increased risk of bleeding [101]. In TA the use of antiplatelet treatment appears to have a protective effect against ischemic events, while neither anticoagulants, nor corticosteroids/immunosuppressants seem to be able to prevent CVE [102].

A retrospective recent study has shown that patients using statins were less likely to develop GCA compared to patients not using them [103]. Interestingly, a recent association study performed using the World Health Organization database, has suggested that the occurrence of PMR could be correlated with statin use [104].

### Others systemic vasculitis

#### Polyarteritis nodosa

Polyarteritis nodosa (PAN) is a multisystemic necrotizing vasculitis of medium-sized arteries, not associated with glomerulonephritis, nor with ANCA positivity [52]. Few conflicting results about thrombotic events in PAN exist; indeed one study on 285 PAN patients has reported a much lower incidence of VTE compared to AAV patients [72], whilst a more recent Swedish population-based study has suggested an increased risk of thrombotic events [88]; of note, the latter study included subjects with different autoimmune diseases observed from 1964 to 2008, thus it is unclear whether or not patients with MPA were included together with PAN patients. Finally, a certain relation seems to exist between PAN and antiphospholipid antibody syndrome, so complicating the scenario [105].

#### Henoch-Schönlein purpura

Henoch-Schönlein purpura (HSP) is a systemic vasculitis of the small vessels mainly affecting children. Thrombotic events are rare complications of HSP, and to date only case reports are reported [106].

#### Kawasaki disease

Kawasaki disease is a systemic vasculitis and represents the most common cause of acquired heart disease in childhood. Sometimes, despite appropriate treatment with acetylsalicylic acid and intravenous immunoglobulins, coronary aneurysms occur; the formation of a thrombus at this level could lead to vascular occlusion and consequent myocardial infarction [107].

#### Retroperitoneal fibrosis

Retroperitoneal fibrosis (RPF) is a rare fibroinflammatory disorder characterized by the presence of a retroperitoneal mass, that could be associated with abdominal aorta aneurysms and/or vasculitis of the thoracic aorta; RPF could be primary or secondary, mainly to neoplastic or infectious diseases. Venous thrombosis could be a presentation symptom in RPF due to the compression of vascular structures, in particular of the inferior vena cava and iliac veins [108].

## Conclusions

The relationships between both innate and adaptive immunity and coagulation are becoming more evident and nowadays it is quite clear that inflammation and thrombosis (both arterial and venous) are tightly related. Moreover, inflammation-induced thrombosis is considered not only a feature of several autoimmune diseases, such as SLE**,** RA or SS, but also of systemic vasculitis.

Among vasculitides, BS represents the model of thrombosis induced by inflammation, but more recently accumulating data have also demonstrated a significant increase in thrombo-embolic events in patients with both small and large vessel vasculitis syndromes. In particular, EGPA, GPA, and GCA have an increased incidence of both venous and arterial thrombotic events especially during the early and active phases of disease.

These concepts have important consequences in terms of management. Indeed, BS requires immunosuppressive agents for venous or arterial involvement rather than anticoagulation or antiplatelet therapy and one might speculate that also in AAV or LVV an aggressive anti-inflammatory treatment during the active phases could improve vascular involvement especially in early stages. Nowadays, conflicting data exist about the role of antiplatelet/anticoagulant therapy in LVV, while their role in AAV is obscure.
